# A novel smart somatosensory wearable assistive device for older adults’ home rehabilitation during the COVID-19 pandemic

**DOI:** 10.3389/fpubh.2023.1026662

**Published:** 2023-09-18

**Authors:** Chien-Hsiang Chang, Wei-Chih Lien, Tseng-Ping Chiu, Tai-Hua Yang, Chun-Chun Wei, Yu-Liang Kuo, Chung-Hsing Yeh, Bo Liu, Pin-Jun Chen, Yang-Cheng Lin

**Affiliations:** ^1^Department of Industrial Design, National Cheng Kung University, Tainan, Taiwan; ^2^Department of Physical Medicine and Rehabilitation, National Cheng Kung University Hospital, College of Medicine, National Cheng Kung University, Tainan, Taiwan; ^3^Department of Physical Medicine and Rehabilitation, College of Medicine, National Cheng Kung University, Tainan, Taiwan; ^4^Department of Orthopaedic Surgery, National Cheng Kung University Hospital, College of Medicine, National Cheng Kung University, Taipei, Taiwan; ^5^Department of Biomedical Engineering, National Cheng Kung University, Tainan, Taiwan; ^6^Department of Digital Multimedia Design, National Taipei University of Business, Taipei, Taiwan; ^7^Faculty of Information Technology, Monash University, Melbourne, VIC, Australia

**Keywords:** frailty, usability, COVID-19, telemedicine, home rehabilitation, wearable assistive device

## Abstract

**Background:**

Due to the Coronavirus disease 19 (COVID-19) related social distancing measures and health service suspension, physical activity has declined, leading to increased falling risk and disability, and consequently, compromising the older adult health. How to improve the quality of older adult life has become a crucial social issue.

**Objective:**

In traditional rehabilitation, manual and repetitive muscle training cannot identify the patient’s rehabilitation effect, and increasing the willingness to use it is not easy. Therefore, based on the usability perspective, this study aims to develop a novel smart somatosensory wearable assistive device (called SSWAD) combined with wireless surface electromyography (sEMG) and exergame software and hardware technology. The older adult can do knee extension, ankle dorsiflexion, and ankle plantar flexion rehabilitation exercises at home. Meanwhile, sEMG values can be digitally recorded to assist physicians (or professionals) in judgment, treatment, or diagnosis.

**Methods:**

To explore whether the novel SSWAD could improve the older adult willingness to use and motivation for home rehabilitation, 25 frail older adult (12 males and 13 females with an average age of 69.3) perform the rehabilitation program with the SSWAD, followed by completing the system usability scale (SUS) questionnaire and the semi-structured interview for the quantitative and qualitative analyses. In addition, we further investigate whether the factor of gender or prior rehabilitation experience would affect the home rehabilitation willingness or not.

**Results:**

According to the overall SUS score, the novel SSWAD has good overall usability performance (77.70), meaning that the SSWAD makes older adult feel interested and improves their willingness for continuous rehabilitation at home. In addition, the individual item scores of SUS are shown that female older adult with prior rehabilitation experience perform better in “*Learnability*” (*t* = 2.35, *p* = 0.03) and “*Confidence*” (*t* = −3.24, *p* = 0.01). On the contrary, male older adult without rehabilitation experience are more willing to adopt new technologies (*t* = −2.73, *p* = 0.02), and perform better in “*Learnability*” (*t* = 2.18, *p* = 0.04) and “*Confidence*” (*t* = −3.75, *p* < 0.001) with the SSWAD. In addition, the result of the semi-structured interview shows that the operation of the SSWAD is highly flexible, thus reducing older adult burden during the rehabilitation exercise and using them long-term.

**Conclusion:**

This novel SSWAD receives consistently positive feedback regardless of the gender or prior rehabilitation experience of elders. The SSWAD could be used as a novel way of home rehabilitation for elders, especially during the COVID-19 pandemic. Older adult can do rehabilitation exercises at home, and physicians could make proper judgments or adjust suitable treatments online according to the sEMG data, which older adult can know their rehabilitation progress at the same time. Most importantly, older adult do not have to go to the hospital every time for rehabilitation, which significantly reduces time and the risk of infection.

## 1. Introduction

Upon aging, immunity declines, and physical function degenerates, which not only leads to chronic diseases but also renders the older adult to be susceptible to infection and mortality. Due to the current global spread of the Coronavirus disease 19 (COVID-19) pandemic, various disease prevention measures, such as social distancing have been implemented worldwide. Such actions aggravate the health problems of the older adult, including chronic diseases, declining immunity, reduced neurocognitive function and mental health, lack of exercise, and increased risk of death (1). In addition, medical services have been suspended due to the COVID-19 pandemic, leading to reduced healthcare monitoring for the older adult. They are also no longer able to perform sufficient exercise, leading to an increase in the incidence of degenerative bone and joint diseases and the deterioration of these illnesses (2). Even in healthy and active elders, a significant increase in the prevalence of frailty is observed due to various factors, such as prolonged stay-at-home, reduced physical activity, sedentary lifestyle, and thus increasing body fat and loss of muscle mass (3). Notably, the consequence of insufficient exercise is concerning among older adult with infections as it creates a wide range of clinical symptoms and complications and an increased risk of sarcopenia (4). With the development of new public health policies and measures, including the growth of telemedicine, the United States healthcare system has experienced significant transformations. To improve healthcare access and reduce infection risk, telemedicine has become a novel healthcare model during the COVID-19 pandemic and for the future (5). Telemedicine not only provides the older adult with the necessary care and information to improve their health but also allows them to be acquainted with an e-health upgrade requirement for a post-pandemic aging society.

Wearable devices have been promoted by advances in wireless sensing technology (6). Meanwhile, pandemic-related lifestyle transitions in recent years have also accelerated the transformation of the conventional healthcare model. One of the goals of telemedicine is to integrate software and hardware technology based on wireless surface electromyography (sEMG) sensing technology to allow medical professionals to personalize the rehabilitation program for patients remotely. In this regard, wearable devices equipped with sEMG sensing technology and rehabilitation gaming systems are essential. Promoting new technologies such as e-health and smart sensing has maximized the benefits of smart wearable devices in healthcare (7). The new norms of COVID-19 have also increased the demand for wearable and Internet of Things (IoT) devices in healthcare. As such, the growth of the wearable device market, which is expected to expand at a compound annual growth rate of 18% between 2021 and 2026, has been accelerated (8).

Furthermore, wearable devices currently allow 24-h monitoring of our comprehensive physiological data, including heart rate, blood oxygen, and sleep status, and consequently the production of a multi-dimensional health database for preventive health services. With its improved accuracy, wearable devices have become a standard effective monitoring tool to provide medical professionals with health data for disease diagnosis and follow-up (9, 10). For example, wearable devices can accurately measure the heart rhythm to identify the risk of arrhythmia and atrial fibrillation, thereby improving the accuracy of cardiovascular disease diagnosis (11). In our aging society, the optimal e-health model for the older adult requires wearable devices in healthcare services and products. At the same time, the acceptance and utilization rate of new technologies among the older adult can be improved by technology education. In addition, product sustainability among the older adult can be enhanced via psychological and emotional guidance, epistemic values, and product quality (12).

This study aims to develop a novel smart somatosensory wearable assistive device (called SSWAD), combined with sEMG and exergame software and hardware technology for rehabilitating the lower limb muscles of the older adult (13–15). Moreover, it’s ergonomic (user-friendly) design could improve the ease of use and reduce the operational burden for elders, thereby promoting their satisfaction with the SSWAD and continuous use for long-term rehabilitation. In other words, the motivation of this study is to provide older adult with a novel rehabilitation tool and a user-friendly care model. While older adult use the SSWAD to do knee extension, ankle dorsiflexion, and ankle plantar flexion rehabilitation exercises, the sEMG values can be digitally recorded to help physicians in judgment, treatment or diagnosis, to improve their health and well-being under the new living norms of the COVID-19 pandemic. The following sections will present a literature review related to wearable devices, the design process of the SSWAD, usability experiments, and discussions of the system usability scale (SUS) results to explore the willingness and motivation for home rehabilitation among the older adult with frailty.

## 2. Literature review

This section presents a literature review on the e-health system, wearable devices for the older adult, market trends, and the significance of functional fitness for the older adult. This section also discusses the status and the design principle of somatosensory game rehabilitation for frail elders, which is used as the basis for developing a novel wearable device in subsequent sections of this study.

### 2.1. E-health system

The decline of multiple physiological functions often accompanies the aging process. With the transformation of social and demographic structures, it has become challenging for the older adult to maintain their quality of life (QoL). Because frailty not only increases the burden of healthcare but also elevates the risk of functional decline and mortality among the older adult, inevitably increasing their healthcare demands each year. Owing to the growing medical and care unmet needs in our aging society, e-health has become an important direction in improving the value of medical services. The development of e-health can bring substantial benefits to the medical industry by reducing travel time to healthcare facilities and the incidence of hospital-acquired infection, even globally (16). In addition, the rapid expansion of the IoT and big data has facilitated the integration and innovation of information and communication technology (ICT) and the healthcare industry, thereby promoting technologies, such as wearable devices and artificial intelligence (AI), to become the development trend of healthcare services (17). In recent years, with the advancement of AI and the application of ICT (such as healthcare, big data, and IoT), multidisciplinary integration between healthcare and ICT has been realized, allowing medical professionals to utilize innovative data analysis techniques in processing and facilitating medical knowledge. In addition, these new technologies can assist medical decision-making and reduce possible iatrogenic errors, thereby effectively improving the efficiency and quality of medical services and optimizing the clinical outcome of the entire healthcare system.

With rapid changes in the global medical industry, the e-health system can improve the existing workflow, efficiency, and accuracy of the healthcare system, thereby facilitating the intelligentization of healthcare services. As a novel type of healthcare, e-health, and telemedicine provide precise and personalized medical services through behavioral science theories and gamification, thereby increasing users’ motivation for self-management and long-term participation (18, 19). The Veterans Health Information Systems and Technology Architecture (VISTA) system constructed by the U.S. Department of Veterans Affairs includes a complete electronic health record system. It provides video-based telemedicine services via videos with similar efficacy to that of conventional healthcare (20). The implementation of e-health not only reduces medical costs but also promotes the transformation of medical services and adds value to the healthcare system. However, while e-health can boost the efficiency of the healthcare system and provide integrated medical services, the adoption and acceptance of e-health remain controversial, thereby presenting some challenges to its development. Van der Kleij (21) proposes three future challenges for e-health, including (1) E-health must provide personalized services for patients; (2) The implementation of e-health must consider the ethics, safety, and privacy of patients; and (3) System design and development must consider the operability of disadvantaged groups and technological illiterates.

### 2.2. Wearable devices for the older adult and the market trends

As the physiological functions of the older adult decline with increasing age, their demand for self-health management increases. Meanwhile, the care for the older adult has increased the expenditure burden of healthcare, long-term care, and social welfare systems. Wearable devices have a wide range of applications in the medical field, including monitoring patients’ acute or chronic diseases and general health conditions, both inside and outside the clinical environment, and providing real-time automated medical services within time and space constraints (22). By incorporating new technological applications and innovations, wearable devices are now capable of delivering remote video and medical monitoring as well as collecting and managing personal physiological data. These data are then transferred back to the medical institution to assist with managing the medical care process. Due to these functions, wearable devices have become the development trend in the healthcare industry. Wearable devices not only innovate and improve the medical care process but also significantly influence the users’ psychological functions. When applied to the older adult, they can effectively encourage and motivate the users (23). A study has indicated that through wearable devices, users can gain a deep understanding of their health status and, consequently, are more willing to take health-promoting measures, thereby enhancing their motivation to participate in learning and expand social activities (24). By combining medicine with ergonomics through innovative technology, wearable devices can serve as daily assistive products for the older adult (25–27). By helping them maintain physical functions, participate in learning, and engage in social activities, wearable devices can improve the autonomy of the older adult and reduce the care burden on families and society, benefiting the national economy (28, 29).

Wearable devices designed for the older adult are among the products with the highest development potential. Among different types of wearable devices, those that provide health and home monitoring have been the focus of various industries. The ultimate goal is to make wearable devices capable of collecting physiological data, improving service processes, and ultimately establishing a comprehensive ecosystem (15, 19). Wearable devices have now been applied to 11 medical fields, including older adult care monitoring, monitoring chronic diseases, activity recognition (monitoring users’ daily activities), clinical applications, emergencies (providing emergency services), mental health, movement disorders, rehabilitation, preventive measures, telemedicine, and paramedicine (30). In particular, the design of wearable devices for the older adult has increasingly focused on the balance between the user interface and the device-wearing form to improve the older adult’s willingness to use the product. Moore et al. (31) propose that the design criteria of wearable devices could be divided into motivational factors, device functions, and ease of use. In addition, the device should maintain a balance between functionality and user experience, prioritizing promoting users’ motivation and social interaction. While wearable devices in the medical monitoring of the older adult facilitate precise care and reduce medical costs, some limitations and challenges remain. One of the challenges is for the older adult to correctly put on the device, which can be resolved by developing software and hardware with high usability to create a balance between user experience and other aspects, such as device function and signal accuracy (32). Furthermore, users’ perception of the wearable device highly depends on their emotional or psychological state. Therefore, wearable devices can improve users’ willingness to use by focusing on their emotions and the ability to attract users, which can be achieved through gamification (12).

### 2.3. Significance of functional fitness of the older adult

With advances in science, technology, and medicine, the average life expectancy of the global population is increasing each year. Therefore, the lifestyle, physiological evaluation, and medical needs of the older adult have become an essential societal issue (29). Moreover, the older adult’s body functions deteriorate, and their physical activities gradually decline, thus resulting in various complications, including abnormal cognitive and sensory functions, gait instability, loss of muscle strength, etc. Therefore, frailty has become a common health issue among the older adult in our society. It has been reported that the older adult’s physical function declines at a steady rate of 1% per year (33). Frailty is a clinical syndrome that results from the interaction between aging and chronic diseases. It affects multiple physiological systems, such as endocrine, cardiovascular, and skeletal muscle systems, which in turn impairs the physiological functions of the older adult and increases the incidence of disability and other adverse events (34). During the COVID-19 pandemic, older adult have become a susceptible population to infection and death. Frailty has been identified as one of the risk factors for severe clinical manifestations. In addition, as the frequency of physical activities of the older adult decreases during the pandemic, the incidence of disability will correspondingly increase in the future and become a significant challenge in the post-pandemic era (35). Therefore, in response to the impact of an aging society, we should focus on addressing the health issues of the older adult. The aging of the older adult not only causes declining physical functions and compromised QoL but also imposes a heavy care burden on families and society.

Declines in both the quality and the strength of the muscle accelerate the rate of frailty and, consequently, the impairment of gait and balance, thereby decreasing mobility and increasing the risk of disability. The interaction of physiological aging and chronic diseases can indirectly or directly induce frailty, further aggravating diseases, sarcopenia, and related conditions, creating a vicious circle of health conditions in the older adult that eventually leads to disability (27). The most effective method against sarcopenia is maintaining regular exercise or a dynamic lifestyle. In addition to supporting body functions, regular exercise can also preserve or improve the coordination and balanced function of the body while slowing down the deterioration of frailty, thereby reducing the risk of disability. A study also points out that functional exercise could delay the decline of the older adult’s physical functions, thereby decreasing the risk of death and fall-related injuries while improving the older adult’s physical function and cognitive ability (19, 36). When combined with proper nutrition support and other physical and mental training that helps maintain a positive mood, functional exercise can reduce the incidence of frailty among the older adult, thereby facilitating them to enjoy an active and happy life. Multimodal training interventions that consist of exercise training, protein supplementation, and other supports have been recommended as the best healthcare strategy for preventing and treating frailty and sarcopenia (37).

## 3. Methods

**Figure 1:**
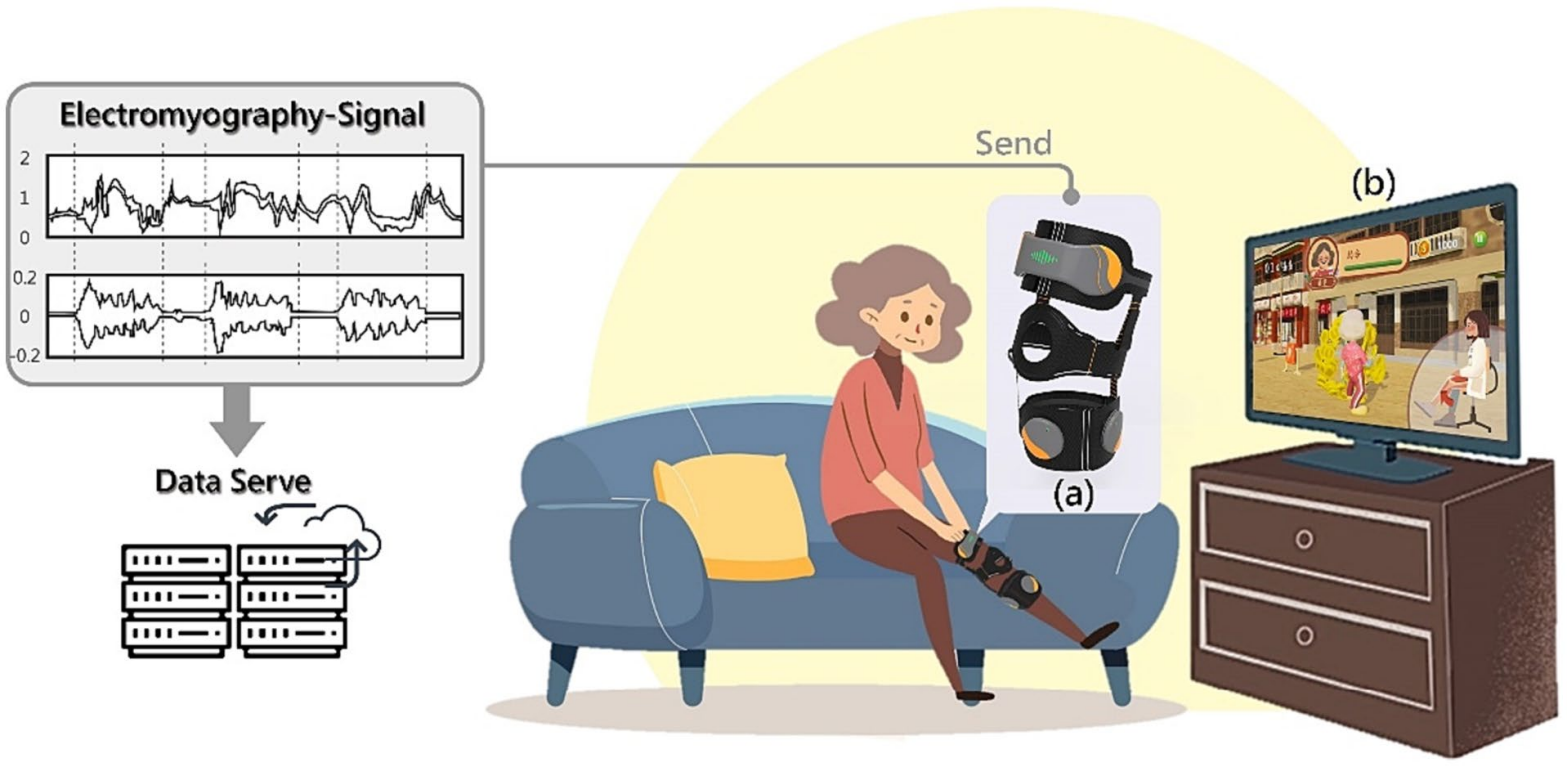
The SSWAD could help frail older adult do rehabilitation exercises at home.

This study aims to develop a user-friendly SSWAD for frail older adult doing rehabilitation exercises at home (as shown in Figure 1). The SSWAD is combined with sEMG (as shown in Figure 1A) and exergame software and hardware technology (as shown in Figure 1B). While older adult do knee extension, ankle dorsiflexion, and ankle plantar flexion exercises, the sEMG values can be digitally recorded to help doctors in judgment, treatment or diagnosis. This section presents the design process and the clinical experiment of the SSWAD.

### 3.1. Design of the smart somatosensory wearable assistive device

The smart somatosensory wearable assistive device (SSWAD) proposed in this study utilizes low-noise sensing elements that could capture sEMG signals to detect the activities of various muscle groups during rehabilitation exercise training. The SSWAD integrates ICT and uses exergame rehabilitation to improve older adult motivation for muscle strength training. Subsequently, the SSWAD is optimized for clinical rehabilitation that could prevent and improve degenerative diseases, such as frailty and sarcopenia. The following sections provide in-depth discussions on the three major aspects of the product design, including hardware and software integration, design process, and final design.

#### 3.1.1. (A) Hardware and software integration

**Figure 2:**
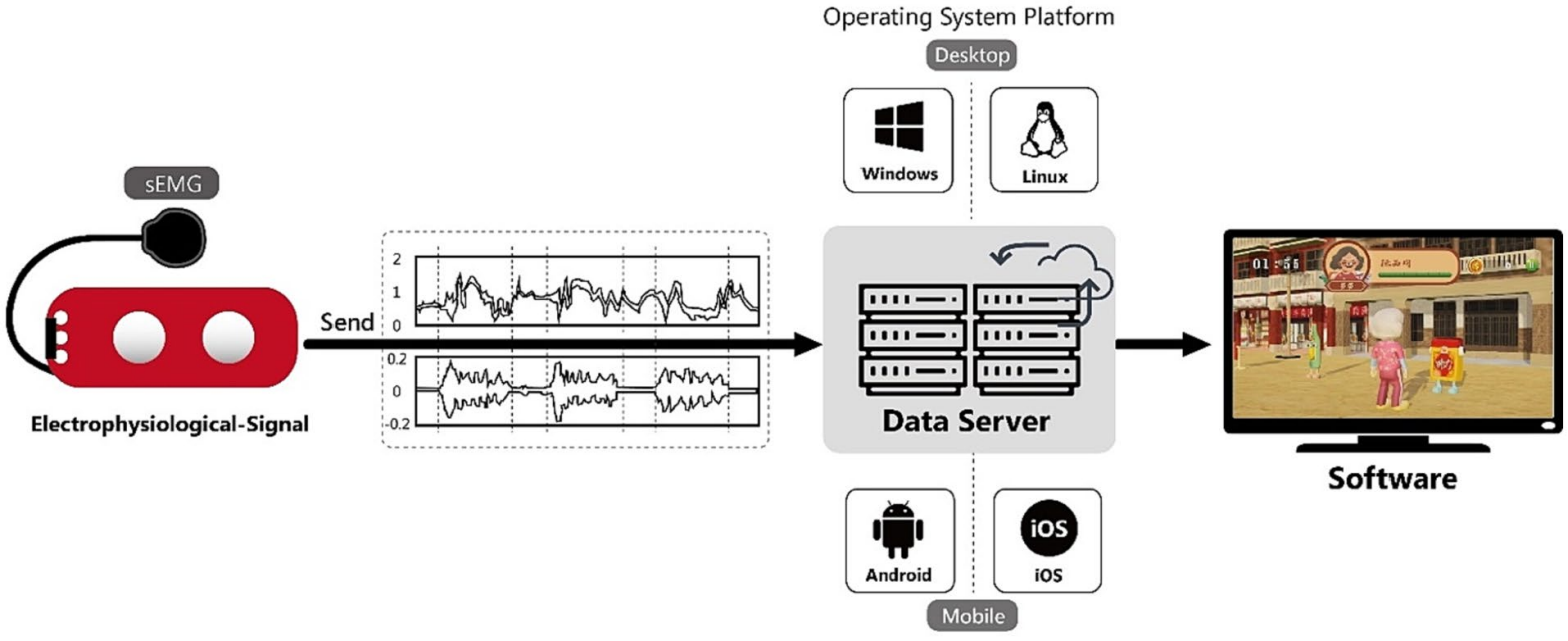
Illustration of hardware and software integration.

The sensor elements of the proposed wearable device, developed with commercially available modules, consist of electrode patches and sEMG sensors. The sEMG sensors measure the performance of the joint operation of muscles and nerves. Together with electrode patches on the surface of the skin, they can detect weak potential differences generated by muscle contraction on the skin surface and collect the signal through the sensing unit. Subsequently, the collected signals are amplified, processed in the amplifying team, and sent to the host so that data can be displayed on the screen in real-time. Meanwhile, relevant signals are stored in the host and can be accessed immediately. The stored signals are then transferred to the Unity game software, which serves as an interactive multimedia medium to provide users with visual feedback (as shown in Figure 2).

#### 3.1.2. (B) The design process of the SSWAD

**Figure 3:**
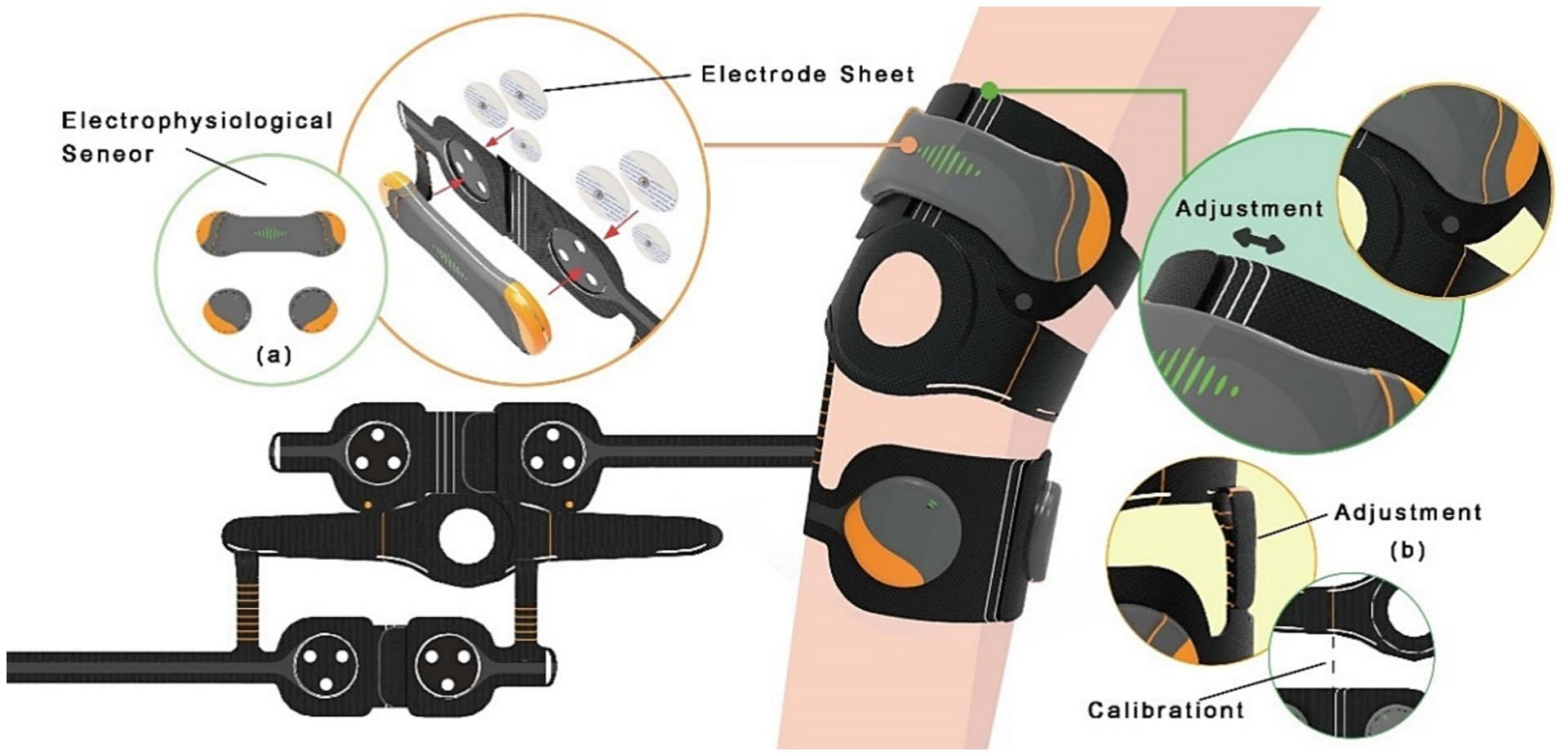
The design of the SSWAD with **(A)** the sensors and **(B)** detachable adjustment belt.

Improvements made to the product according to the priorities determined from expert interviews and user tests, a novel SSWAD is developed with a circuit board to capture low-noise sEMG signals for detecting the motion of various muscle groups during training. The product is featured a human-oriented design and could be customized. The device is made of fabric material and is in direct contact with the skin during usage, requiring regular cleaning when worn for an extended period. Therefore, the sensors are designed to be detachable. In addition, the sensor hardware is made with a droplet shape to guide the user to attach sensors to the wearable device with their sharp ends facing outwards so that the three buttons at the bottom of the sensor could accurately align with the three small round engagement holes on the wearable device to calibrate. In addition, the sensors are connected to the wearable assistive device using hook and loop fasteners to improve the ease of wearing (as shown in Figure 3A).

The SSWAD consists of two detachable parts, which not only facilitates position adjustment to a great extent but also allows users to adjust and calibrate the wearing position according to their specific self-rehabilitation needs. Standard clinical rehabilitation programs include three major movements, i.e., knee extension, ankle dorsiflexion, and ankle plantar flexion. When the user is performing knee extension, they only need to put on the top half of the knee pad, while the bottom half could be removed to reduce the user burden (as shown in Figure 3B). In addition, the middle hole is used as a calibration point, which should be aligned with the knee. During the first attempt, the user is assisted by medical personnel in wearing the device and adjusting its position. In subsequent uses, the user needs to align the middle hole of the knee pad to the center point of the knee, to ensure the right position, before starting rehabilitation.

**Figure 4:**
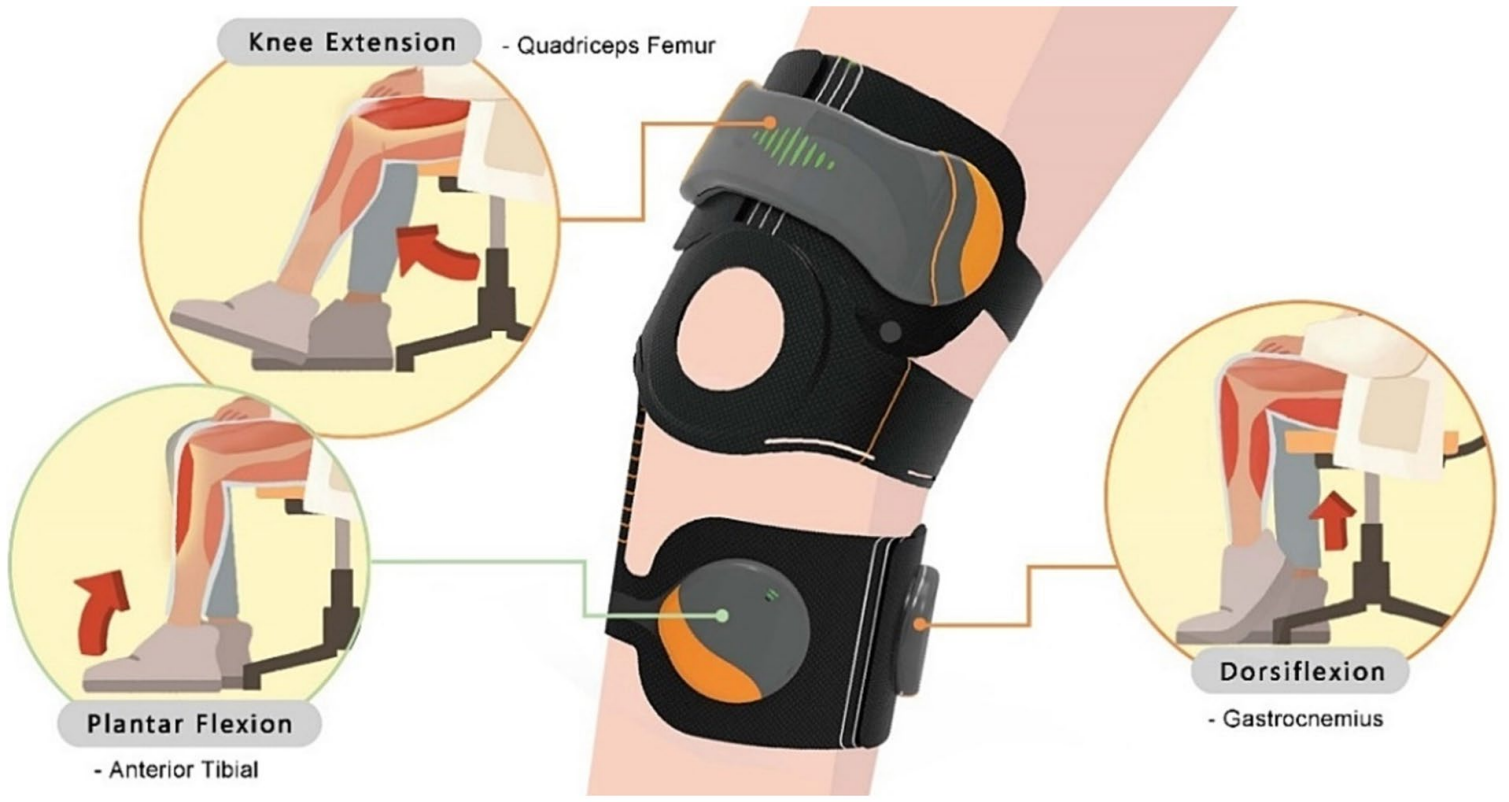
The corresponding relationship between rehabilitation movements and muscle groups. For example, the “knee extension” corresponds to the “quadriceps femoris,” the “ankle dorsiflexion” is training the “gastrocnemius,” and the “ankle plantar flexion” is used the “tibialis anterior” muscle.

However, this study’s wearable rehabilitation device is mainly designed to train lower limb muscles. These muscles can be divided into quadriceps femoris, tibialis anterior, gastrocnemius, etc. Their locations are on the left and right sides three fingers above the knee, the medial-lateral aspect of the tibia, and the calf, respectively (as shown in Figure 4). To ensure the alignment of the sEMG sensors with the optimal sensing position of the target muscle group, the connection between the upper and lower parts of the device is made adjustable by using hook and loop fasteners. As such, the size of the device and the sensor positions are flexible. In addition, the wearing position is recorded on the device with a scale line to make it easier for the user to locate the optimal muscle sensing position when putting the device on next time, thereby improving the device’s wearability.

### 3.2. Clinical experiment of the SSWAD

The clinical experiment is performed to collect quantitative and qualitative data to explore the usability and feasibility of the SSWAD developed in this study.

#### 3.2.1. (A) Subjects

**Figure 5:**
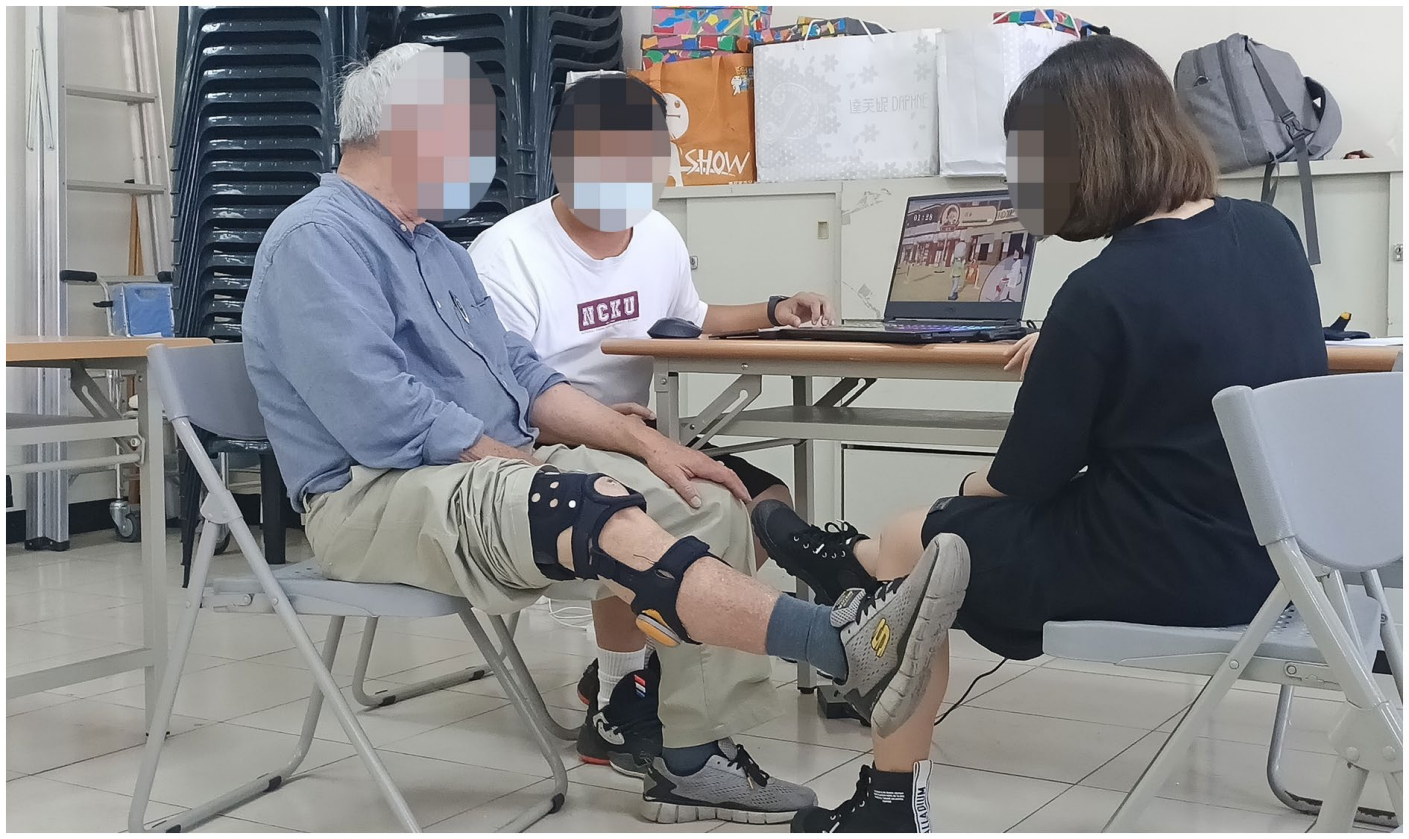
An older adult and two researchers for the clinical experiment.

This study recruits 25 subjects (12 males and 13 females) from a community activity center in Tainan, Taiwan. They are between 60 and 83 years of age (with an average of 69.3 years), and attend social activities 2–3 times per week. In addition, 16 (8 males and 8 females) have no rehabilitation experience, whereas the remaining 9 (4 males and 5 females) have prior rehabilitation experience. All subjects have the ability to perform simple movements with their lower limbs, have normal cognitive ability, and are able to clearly express their thoughts. The recruitment criteria are as follows: (1) Older adult could be with a clinical frailty scale of 4–6 (38); (2) Older adult could perform simple actions as required; (3) Older adult could demonstrate an average cognitive ability; (4) Older adult could cooperate with instructions from the researchers and the exergames. In addition, the subjects are required to provide subjective evaluations of the experiment upon its completion. Since the subjects are older adult aged above 60 years, a medical staff and two researchers are present during the experiment to assist them in completing the experiment, as shown in Figure 5.

#### 3.2.2. (B) The procedure of the clinical experiment

**Figure 6:**
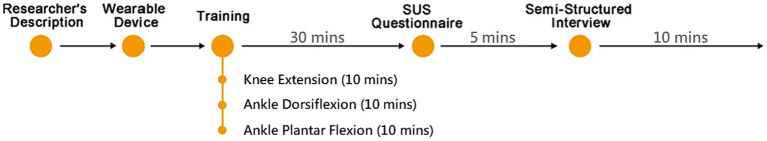
The experimental flow chart.

Before the experiment began, researchers first explained the system operation and wearing instructions of the wearable device to the older adult and helped them adjust the size of the device. Subsequently, the older adult were left to operate the system and put on the SSWAD independently. During the experiment, after the assistive device was put on, the older adult would immediately turn on the system to perform systematic rehabilitation training. The system would then guide the older adult to randomly perform one of the three major rehabilitation exercises for lower limbs, including knee extension, ankle plantar flexion, and ankle dorsiflexion, each with a duration of 10 min (a total of 30 min of training). After the rehabilitation training, a five-point SUS questionnaire was completed to collect the older adult perceptions of the SSWAD, during which their verbal responses were recorded by the researchers. In addition, semi-structured interviews were conducted to understand the older adult subjective feelings and willingness to use the device, as well as any issues they encountered during the operation. The length of the interview was approximately 15 min for each elder (as shown in Figure 6). The questionnaire was modified from the SUS (39), including 10 questions: *Willingness* (*I1*), *Complexity* (*I2*), *Convenience* (*I3*), *Stress* (*I4*), *Integration* (*I5*), *Inconsistency* (*I6*), *Learnability* (*I7*), *Cumbersomeness* (*I8*), *Confidence* (*I9*), and *Difficulty* (*I10*) (as shown in Table 1). Each question was scored on the five-point Likert scale (with 1 representing “Strongly disagree” and 5 representing “Strongly agree”).

**Table 1:** The system usability scale (SUS) questionnaire.

Item		Description
1.	Willingness	I think that I would like to use this smart somatosensory wearable assistive device (SSWAD) frequently.
2.	Complexity	I found the SSWAD unnecessarily complex.
3.	Convenience	I thought the SSWAD was easy to use.
4.	Stress	I think that I would need the support of a technical person to be able to use this SSWAD.
5.	Integration	I found the various functions in this SSWAD were well integrated.
6.	Inconsistency	I thought there was too much inconsistency in this SSWAD.
7.	Learnability	I would imagine that most people would learn to use this SSWAD very quickly.
8.	Cumbersomeness	I found the SSWAD very cumbersome to use.
9.	Confidence	I felt very confident using the SSWAD.
10.	Difficulty	I needed to learn a lot of things before I could get going with this SSWAD.

## 4. Results

A five-point SUS questionnaire is used to investigate whether the factor of gender or prior rehabilitation experience would affect older adult home rehabilitation willingness or not.

### 4.1. The usability score of the SSWAD

Investigation of the usability of the wearable device for older adult is carried out mainly via a SUS questionnaire to understand the older adult perceptions of 10 aspects of the SSWAD (as shown in Table 1). Subsequently, the t-test is adopted to verify the correlation between usability and elder gender or prior rehabilitation experience. The responses to the SUS questionnaire are listed in Table 2. From Table 2, the overall SUS score for the SSWAD is 77.70, indicating that the SSWAD has a “Good” overall usability performance (19, 40, 41).

**Table 2:** The overall SUS scores for the SSWAD.

Subject	Gender	Prior rehab experience: Yes or No	SUS Score	Mean (SD)
1	Female	Yes	62.50	77.70 (12.22) Good
2	Female	Yes	97.50	
3	Female	Yes	92.50	
4	Female	Yes	70.00	
5	Female	Yes	77.50	
6	Female	No	72.50	
7	Female	No	65.00	
8	Female	No	65.00	
9	Female	No	90.00	
10	Female	No	95.00	
11	Female	No	67.50	
12	Female	No	60.00	
13	Female	No	80.00	
14	Male	Yes	72.50	
15	Male	Yes	82.50	
16	Male	Yes	50.00	
17	Male	Yes	65.00	
18	Male	No	82.50	
19	Male	No	90.00	
20	Male	No	77.50	
21	Male	No	85.00	
22	Male	No	85.00	
23	Male	No	85.00	
24	Male	No	87.50	
25	Male	No	85.00	

To further investigate, Table 3 shows the individual item benchmarks for the SSWAD. For the SSWAD, there are six items (out of 10 items) that achieve the “*Good*” benchmark (including *Good ^–^* and *Good^+^*) (*Items 1–5* and *Item 9*) (40, 41), while the other four items reach the “*Average*” level (including *Average^−^* and *Average^+^*). It responds to the previous result that the overall SUS score of the SSWAD has “*Good*” usability performance. From Table 3a, it is worth mentioning that *Item 7* (*Learnability*) for “Female” only gets the “*Poor*” benchmark (the single item score being 3.46), thus suggesting that there is much room for improvement in the “*Learnability*” for the female elders. To further examine the effects of prior rehabilitation experience and gender, the following t-test analyses are conducted.

**Table 3:** Basic descriptive statistics and item benchmarks for SUS.

Item	(a) Gender (1 is the lowest, 5 is the highest)		(b) Prior rehab experience (1 is the lowest, 5 is the highest)		(c) SSWAD	
	Male M (SD)/*n* = 12	Item benchmark	Yes M (SD)/*n* = 9	Item benchmark	Mean (SD)/*n* = 25	Item benchmark
	Female M (SD)/*n* = 13		No M (SD)/*n* = 16			
1	4.00 (1.13)	Good^+^	3.33 (1.22)	Average	3.96 (1.09)	Good
	3.92 (1.11)	Good	4.31 (0.87)	Good^+^		
2	2.00 (1.09)	Good^−^	2.33 (1.41)	Average^+^	1.96 (1.17)	Good^−^
	1.92 (1.26)	Good	1.75 (1.00)	Good^+^		
3	4.41 (1.16)	Good^+^	4.11 (1.27)	Good^−^	4.44 (1.04)	Good^+^
	4.46 (0.96)	Good^+^	4.50 (1.10)	Good^+^		
4	1.41 (0.67)	Good^+^	1.89 (1.27)	Average	1.48 (0.92)	Good
	1.53 (1.12)	Good	1.56 (1.09)	Good		
5	4.17 (0.71)	Good^+^	4.11 (0.78)	Good^+^	4.12 (0.83)	Good^+^
	4.07 (0.95)	Good^+^	4.12 (0.88)	Good^+^		
6	2.25 (0.62)	Average	2.22 (0.97)	Average	2.28 (0.94)	Average^−^
	2.30 (1.18)	Average^−^	2.31 (0.94)	Average^−^		
7	4.08 (1.00)	Good^−^	4.11 (0.78)	Good^−^	3.76 (1.05)	Average^+^
	3.46 (1.05)	Poor	3.56 (1.15)	Average^−^		
8	1.91 (0.90)	Good^−^	2.33 (1.41)	Average^−^	2.00 (1.12)	Average^+^
	2.07 (1.32)	Average^+^	1.81 (0.91)	Good^−^		
9	4.25 (0.97)	Good	4.11 (1.05)	Good^−^	4.48 (0.82)	Good^+^
	4.69 (0.63)	Good^+^	4.69 (0.60)	Good^+^		
10	1.75 (0.97)	Good^−^	2.00 (1.00)	Average^+^	1.96 (1.09)	Average^+^
	2.15 (1.21)	Average	1.93 (1.18)	Average^+^		

### 4.2. Analysis of the gender factor

This section explores the effect of gender on the acceptance of the novel SSWAD developed in this study. From Table 4a, regardless of their gender, the older adult responses to the SUS questionnaire are consistent (no significant difference) after using the SSWAD (*p* > 0.05). For example, for *Item 1* (*Willingness*) of the SUS questionnaire, “*I think I would like to use this Smart Somatosensory Wearable Assistive Device (SSWAD) frequently*,” the t value is 0.17 (*p* = 0.86). Similarly, for *Item 3* (*Convenience*), “*I thought the SSWAD was easy to use*,” the t value is-0.11 (*p* = 0.92). The results suggest that the subjective feelings of both males and females regarding the use of the SSWAD for rehabilitation are no significant difference.

**Table 4:** The *t*-test for the effect of the gender factor.

Item	(a)	(b)	(c)
	Gender *t* value/*p* value	With prior rehab experience *t* value/*p* value	Without rehab experience *t* value/*p* value
	(Twelve males and thirteen females)	(Four males and Five females)	(Eight males and Eight females)
1	0.17/0.86	−0.71/0.50	0.85/0.41
2	0.16/0.87	0.77/0.47	−0.48/0.63
3	−0.11/0.92	−0.22/0.83	−0.44/0.66
4	−0.32/0.74	2.22/0.06	−0.67/0.51
5	0.26/0.79	−1.28/0.24	1.14/0.27
6	−0.15/0.88	0.74/0.48	−0.78/0.45
7	1.52/0.14	−0.36/0.73	2.18/0.04*
8	−0.35/0.72	0.29/0.78	−0.81/0.43
9	−1.36/0.18	−3.24/0.01*	0.40/0.69
10	−0.92/0.36	0.64/0.54	−1.54/0.14

The symbol * means the *p* value < 0.05, indicating that has a significant difference.

To provide in-depth discussions, further gender analysis is carried out by stratifying older adult into those with prior rehabilitation experience and those without rehabilitation experience. Table 4b shows the result of the older adult with prior rehabilitation experience. For *Item 9* (*Confidence*), “*I felt very confident using the SSWAD*,” the result shows a significant difference between different genders (*t* = −3.24, *p* = 0.01). In contrast, according to *Item 7* (*Learnability*) in Table 4c, for older adult without rehabilitation experience, the result is substantially different between different genders (*t* = 2.18, *p* = 0.04).

In summary, the results of *Item 1* (*Willingness*)(*t* = −0.71, *p* = 0.50 for with prior rehabilitation experience; *t* = 0.85, *p* = 0.41 for without rehabilitation experience) and *Item 3* (*Convenience*)(*t* = −0.22, *p* = 0.83 for with prior rehabilitation experience; *t* = −0.44, *p* = 0.66 for without rehabilitation experience) show they are not affected by the gender factor. However, significant gender differences are observed in the “*Confidence*” level (*Item 9*) for older adult with prior rehabilitation experience and in the “*Learnability*” level (*Item 7*) for those without rehabilitation experience. In this regard, female older adult with prior rehabilitation experience are likelier to master new products (or systems), and male older adult without rehabilitation experience are easier to learn new products (or systems).

### 4.3. Analysis of the prior rehabilitation experience factor

This section is to identify the acceptance of the novel SSWAD among the frail older adult who have participated in traditional rehabilitation for lower limbs and to explore whether prior rehabilitation experience would affect the willingness to use the SSWAD for rehabilitation. The t-test analysis is performed between older adult with and without prior rehabilitation experience as well as between older adult of different genders, as shown in Table 5. From Table 5a, it can be seen that regardless of prior experience, older adult responses to *Item 4* (*Stress*), “*I think that I would need the support of a technical person to be able to use this SSWAD*” (*t* = −0.59, *p* = 0.56), and *Item 8* (*Cumbersomeness*), “*I found the SSWAD very cumbersome to use*.” (*t* = 1.12, *p* = 0.27), are no significant differences. These results reveal that both the ability to use and the perceived complexity of the SSWAD are consistent between older adult with and without rehabilitation experience. In other words, neither the usability nor the operational complexity of the SSWAD would introduce additional pressure or rejection among the older adult regardless of prior rehabilitation experience. By contrast, for *Item 1* (*Willingness*), the t value is-2.32 (*p* = 0.02), suggesting that in terms of “*willingness to use*,” older adult without rehabilitation experience are more willing to continuously use the SSWAD than those with prior rehabilitation experience. This is likely because older adult without rehabilitation experience are willing to try easy-to-access wearable rehabilitation devices.

**Table 5:** The t-test for the effect of the prior rehabilitation experience factor.

Item	(a)	(b)	(c)
	Rehab experience *t* value/*p* value	Males *t* value/*p* value	Females *t* value/*p* value
	(Nine with rehab experience and sixteen without)	(Four with rehab experience and eight without)	(Five with rehab experience and eight without)
1	−2.32/0.02*	−2.73/0.02*	−0.81/0.43
2	1.21/0.24	1.78/0.10	0.16/0.87
3	−0.37/0.71	0.16/0.87	−0.76/0.46
4	−0.59/0.56	0.29/0.78	−0.85/0.41
5	−0.03/0.97	−1.50/0.16	0.96/0.36
6	−0.23/0.82	0.98/0.35	−0.73/0.48
7	1.26/0.22	−0.19/0.85	2.35/0.03*
8	1.12/0.27	1.72/0.12	0.25/0.80
9	−1.75/0.09	−3.75/0.00*	0.47/0.65
10	0.13/0.89	1.31/0.22	−0.81/0.43

The symbol * means the *p* value < 0.05, indicating that has a significant difference.

To further investigate, the same gender reveals that male older adult respond to *Item 1* (*Willingness*) (*t* = −2.73, p = 0.02) and *Item 9* (*Confidence*) (*t* = −3.75, *p* < 0.001) having significant differences between them with and without prior rehabilitation experience (Table 5b). This finding indicates that in terms of willingness to use and confidence level, male older adult without rehabilitation experience feel it easier to master the SSWAD, thereby increasing their intention to use it in the long term.

Alternatively, female older adult respond to *Item 7* (*Learnability*) (*t* = 2.35, *p* = 0.03) having a significant difference between them with and without rehabilitation experience (Table 5c). The result shows that female older adult with prior rehabilitation experience demonstrated high learnability toward the SSWAD, suggesting that prior rehabilitation experience significantly impacted the female older adult learnability of the SSWAD. In particular, female older adult with rehabilitation experience find it easier to learn and operate as their prior experience in clinical rehabilitation training escalated their learnability toward the device.

### 4.4. Two-way ANOVA on the willingness, learnability, and confidence items

For further analyses, we use the two-way ANOVA (42) to explore the relationship between the gender and prior rehabilitation experience factors, and their interactions on *Item 1 (Willingness)*, *Item 7* (*Learnability*), and *Item 9 (Confidence)* according to the previous results. For example, is there any interaction between the gender and the prior rehabilitation experience on the dependent variable (i.e., the SUS score, such as *Willingness*, *Learnability*, or *Confidence*)?

Table 6 shows whether a significant interaction exists or not. If there is a statistically significant interaction, we need to further perform “simple main effects.” Alternatively, if not, we should report “main effects.” Table 6 shows that a statistically significant interaction exists on *Item 9* (*Confidence*), suggesting that no matter what the gender is or prior rehabilitation experience is, it will influence older adult confidence. It is consistent with the results presented in Sections 4.2 and 4.3. In addition, a simple main effect is used to examine why an interaction exists and what causes the interaction happened (42). A simple main effect is a ‘main effect’ of one factor (e.g., the gender factor) at a given level of a second factor (e.g., the prior rehabilitation experience). Please refers to Sections 4.2 and 4.3 for the result of the simple main effects.

**Table 6:** The two-way ANOVA on the willingness, learnability, and confidence items.

	Item 1 (Willingness)		Item 7 (Learnability)		Item 9 (Confidence)	
	*F* value	*p* value	*F* value	*p* value	*F* value	*p* value
Gender * Rehabilitation	0.76	0.39	2.68	0.12	7.75	0.01*
Gender	0.00	0.98	1.31	0.27	7.75	0.01*
Prior Rehabilitation Experience	4.54	0.05*	1.76	0.20	4.65	0.04*

The symbol * means the *p* value < 0.05, indicating that has a significant difference.

From Table 6, *Item 1 (Willingness)* (*F* = 4.54, *p* = 0.05) has a statistical significance of the ‘main effect’ on the prior rehabilitation experience. With further analysis, we find strong evidence that older adult without prior rehabilitation experience have a higher willingness (4.31) than those with prior rehabilitation experience (3.33), which is consistent with the results presented in Sections 4.1 and 4.3. The further discussion will be presented in Section 5.

## 5. Discussion

This study aims to develop a user-friendly SSWAD for frail older adult doing rehabilitation exercises at home. This section will provide in-depth discussions of the quantitative analysis of the usability of the SSWAD as well as the qualitative analysis of semi-structured interviews.

In the analysis of the gender factor, the results show that the subjective feelings of both males and females regarding the use of the SSWAD for rehabilitation are no significant difference. While the pathophysiology that results in frailty is not well identified, an individual’s gender appears to be an important factor influencing the aging trajectory. Gender differences in the effectiveness of gamification interventions in frailty have not been addressed in the research literature to date (43). This finding might be explained by the fact that the novel SSWAD does not introduce additional pressure and inconvenience to rehabilitation and, consequently, would not compromise older adult willingness for self-rehabilitation and long-term use in frail males and females.

In the further gender analysis, the results show that female older adult with prior rehabilitation experience are more confident in using the novel assistive device, suggesting that they are more capable of mastering new products than male elders. Interestingly, male older adult without rehabilitation experience are more likely to learn to use the new product than females. The male older adult may have a profound experience in digital technology in the past (44), and consequently, they find it simple to learn and use new technology products. This could be explained the previous result why female older adult only get the “*Poor*” benchmark on *Learnability* (Item 7). These findings are worthy to further investigate in the future.

From analyses of the effects of gender and prior rehabilitation experience, older adult generally provide positive feedback on the design of the novel SSWAD. In addition, both the wearing and the operation of the device are simple enough to prevent additional pressure or rejection among the older adult, making it suitable for the general frail older adult population. These results also show that female older adult with prior rehabilitation experience find it easier to learn and master the device, and help them establish their confidence in the device and thus escalate their willingness to use the device in the long term. Interestingly, similar results are obtained from male older adult without rehabilitation experience. With the spread of the COVID-19 pandemic, the traditional medical industry has substantially transformed. Despite their declining physical function, older adult have gradually become acquainted with telemedicine, improving their learning ability of new technologies (19). In particular, this study reveals that male older adult without rehabilitation experience are more receptive to new technologies and more willing to try novel wearable rehabilitation devices and use them long-term (9, 10).

In addition, semi-structured interviews are conducted to understand the frail older adult’s subjective feelings toward using the SSWAD for lower limb rehabilitation. Results of the qualitative analysis show that the operation of the SSWAD is highly flexible as it is made of fabric materials and utilized a detachable design. As a result, the device could reduce user burden during the rehabilitation exercise, resulting in their positive feedback on wearing comfort. In addition, by being equipped with sensors that could detect muscle movement during the rehabilitation exercise, the device could help the older adult understand the outcomes of the exercise. Since the device integrates innovative technology for data transfer and record, it could also provide medical professionals with references for follow-up evaluation and the personalization of the rehabilitation training program. With these advantages, the proposed device in this study could improve the older adult’s acceptance of technological aids and telemedicine during the post-pandemic era, consistent with previous studies’ findings (12, 13, 23).

- The product looks techy, feels interesting, and is comfortable to wear.
- I already have trouble moving around, not to mention going to the hospital for rehabilitation. If such a device is available, it will be our savior.
- I can use the device for home rehabilitation without going to the hospital, which is very convenient.

To make the design of the device more human-oriented, the study also explores difficulties encountered by frail older adult while using the novel SSWAD. It summarizes the older adult requirements for the format and content of lower limb rehabilitation. From the results of the semi-structured interviews, some improvements are identified. For example, the wearable device is designed to be individually adjustable and detachable to reduce the older adult’s burden of wearing it. However, this design also results in multiple steps while putting the device on, and the lack of clear operation instructions could increase the risk of operational errors. Therefore, a fault-proof mechanism is introduced to prevent possible errors in device operation.

- There are so many steps involved in putting on the device, which feels complicated. It is easy to forget how to wear the device without clear instructions.
- I need someone to guide me step by step to be able to use the device.

In summary, the older adult feedback on the wearing comfort of the SSWAD is generally positive. Although the detachable design of the device allows a high degree of operational freedom, it also makes the wearing process relatively complicated, which is consistent with the findings of previous research (14, 15, 31).

## 6. Conclusion

With the advancement of wireless sensing technology and lifestyle transition under the COVID-19 pandemic, wearable devices have emerged as an essential area of the medical industry. By integrating a wearable assistive device for lower limbs that could capture sEMG signals with gaming contents, this study has developed a novel smart somatosensory wearable assistive device (SSWAD) for clinical rehabilitation to help prevent and improve degenerative diseases such as frailty and sarcopenia. 25 older adult with frailty are recruited to test the SSWAD, determine the design criteria of wearable rehabilitation devices, and assess the device’s usability. The effects of gender and prior rehabilitation experience on the device’s usability are explored. In addition, the qualitative interview is performed to understand the older adult subjective perception of the SSWAD and any difficulties they encounter. The result shows that older adult with different genders and different rehabilitation experiences have significant differences in perceived “*Willingness*,” “*Confidence*,” and “*Learnability*” of the SSWAD. In this regard, male older adult without rehabilitation experience are more receptive to the SSWAD. Since the SSWAD is easy to learn and use, the older adult confidence could be improved, resulting in their increased willingness to use the device for rehabilitation continuously.

However, there are some limitations of this study. Firstly, due to the declining physical function of the older adult, visual stimulation elements may need to be introduced to future products to reduce possible user errors and operational complexity. For example, a fault-proof design (such as assigning different parts of the device with different shapes and colors) is recommended to guide the wearing process. Secondly, efforts should be made to achieve an integrated wearing design that features fewer adjustable slots and easier operations. Additionally, other assistive technologies should be considered to optimize the usability for the older adult. Thirdly, the sample size is small (25 subjects), and, for future studies, a larger sample size should be considered to get more precise analysis results. Lastly, there is another limitation the subjects are recruited from a community activity center in Taiwan, which the social context of the older adult and the measures taken during the COVID-19 pandemic in Taiwan are not the same as in other nations. For future studies, it should be verified in different contexts or nations to be extended and generalizable.

In conclusion, the SSWAD developed in this study receives positive feedback from the older adult, due to its appropriate wearability, operational usability, and integrated physiological sensing technology with exergame rehabilitation. Therefore, the SSWAD can serve as a health promotion tool to improve the older adult’s health and well-being. In the future, the health ageing platform could be built so older adult can do rehabilitation exercises at home, physicians could adjust suitable treatments online for them, and older adult can know their rehabilitation progress. It is a virtuous cycle that may represent a promising solution for home rehabilitation or telemedicine.

## Data availability statement

The original contributions presented in the study are included in the article/supplementary material, further inquiries can be directed to the corresponding author/s.

## Ethics statement

The studies involving humans were approved by National Cheng Kung University Human Research Ethics Committee, No. NCKU HREC-F-109-497-2, on 4 Feb 2021. The studies were conducted in accordance with the local legislation and institutional requirements. The participants provided their written informed consent to participate in this study.

## Author contributions

All authors listed have made a substantial, direct, and intellectual contribution to the work and approved it for publication.

## Funding

This research was partly supported by the Ministry of Science and Technology, Taiwan, under Grant nos. MOST 110-2622-H-006-008, MOST 110–2823–8-006-003, and MOST 111-2923-E-006 -002 -MY3.

## Conflict of interest

The authors declare that the research was conducted without any commercial or financial relationships that could be construed as a potential conflict of interest.

## Publisher’s note

All claims expressed in this article are solely those of the authors and do not necessarily represent those of their affiliated organizations, or those of the publisher, the editors and the reviewers. Any product that may be evaluated in this article, or claim that may be made by its manufacturer, is not guaranteed or endorsed by the publisher.
